# COVID-19–Related Trajectories of Psychological Health of Acute Care Healthcare Professionals: A 12-Month Longitudinal Observational Study

**DOI:** 10.3389/fpsyg.2022.900303

**Published:** 2022-06-30

**Authors:** Sandra Abegglen, Robert Greif, Alexander Fuchs, Joana Berger-Estilita

**Affiliations:** ^1^Department of Health Psychology and Behavioural Medicine, University of Bern, Bern, Switzerland; ^2^Department of Anaesthesiology and Pain Medicine, Bern University Hospital (Inselspital), University of Bern, Bern, Switzerland; ^3^School of Medicine, Sigmund Freud University Vienna, Vienna, Austria; ^4^Institute for Medical Education, University of Bern, Bern, Switzerland; ^5^CINTESIS - Center for Health Technology and Services Research, Faculty of Medicine, Porto, Portugal

**Keywords:** COVID-19, acute care, psychological resilience, healthcare workers, mental health

## Abstract

The COVID-19 pandemic hit healthcare professionals (HCPs) hard, potentially leading to mental health deterioration. This longitudinal study investigated the 1-year evolution of psychological health of acute care HCPs during the COVID-19 pandemic and explored possible differences between high and low resilient HCPs. From April 2020 to April 2021, a convenience sample of 520 multinational HCPs completed an online survey every 3 months, up to five times. We used mixed linear models to examine the association between resilience and the variation of COVID-19-related anxiety, depressiveness, perceived vulnerability, and psychological trauma symptomatology. We demonstrated “u-shaped” trajectories for all mental health symptoms. We also explored differences in the abovementioned variables between front-line and second-line acute care HCPs. In contrast to HCP.s with lower levels of resilience (-1SD), those with higher levels of resilience (+1SD) showed increased COVID-19 anxiety and perceived vulnerability over time. Front-line and second-line HCPs differed in their depressiveness and psychological trauma variation during the 1-year analysis. High and average resilient second-line HCPs showed steeper depressiveness increases with time than high and average resilient front-line HCPs. Acute care HCPs reported their most elevated clinical symptoms of depressiveness (5–7%) and psychological trauma symptomatology (26–46%) in April 2020. During the first year of the COVID-19 pandemic, second-line HCPs with more resilience showed a steeper worsening of their depressiveness than more resilient front-line HCPs. HCPs with low resilience may benefit from interventions at the beginning of a pandemic, whereas HCPs with high resilience might benefit from resilience-enhancing interventions at later phases.

**Trial Registration:**

The study protocol was pre-registered with the International Standard Randomised Controlled Trial Number (ISRCTN13694948) published (Fuchs et al., 2020).

## Introduction

The ongoing global coronavirus 2019 (COVID-19) pandemic led to severe disruptions in healthcare systems (World Health Organization, 2021). Many hospitals worldwide faced a surge in patients with COVID-19, while others had to plan and reorganise extensively to avoid being overwhelmed (Lien et al., 2020; NHS England, 2020; World Health Organization, 2020). Acute care healthcare professionals (acHCP) had to adapt to abrupt changes in their working conditions, unfamiliar working spaces due to relocations, new colleagues, and ever-changing personal safety and treatment guidelines (Douillet et al., 2021). The inadequate protection against contamination from patient exposure associated with overwork, frustration regarding anti-vaccination campaigns, disease denial and misinformation, discrimination, isolation and limited family contact further impacted the mental health of acHCPs (Pappa et al., 2020; Serrano-Ripoll et al., 2020).

Anaesthesia and intensive care medicine are specialities at risk for psychological health changes by their high demands (physical and emotional) and stress levels. Both specialities deal with long working hours in high-risk, complex working environments involving multidisciplinary teams (Wong and Olusanya, 2017). AcHCPs (working in intensive care, anaesthesia, and emergency medicine) were the vast majority of front-line HCPs during the COVID-19 pandemic. AcHCPs faced personal protective equipment, medications, and ventilator shortages and needed to care daily for severely ill or dying patients (Greenberg et al., 2015; Magnavita et al., 2020b; Th’ng et al., 2021; Tsan et al., 2021).

From the early stages of the COVID-19 epidemic, several publications raised concerns that front-line HCPs were being affected by post-traumatic stress, anxiety, depressiveness, and burnout (Lai et al., 2020; Cag et al., 2021) In front-line HCPs (defined as HCPs having direct contact with COVID-19–infected patients), the pooled prevalence for anxiety has been estimated to range from 23.2% (Pappa et al., 2020) to 32.0% (Luo et al., 2020) and depression from 22.8% (Pappa et al., 2020) to 28% (Luo et al., 2020). A survey conducted in March–April 2020 on the staff of two Italian hospitals showed that the team’s occupational stress and depression during the first wave of the epidemic were, on average, not higher than those recorded in previous years. However, HCPs who had unprotected contact with COVID-19 patients and those who were SARS-CoV-2 positive had an increased risk of insomnia, anxiety and depression when compared to their colleagues (Magnavita et al., 2020c). Unfortunately, most studies addressing front-line professionals’ mental health are cross-sectional or retrospective and have no control group. Psychic symptoms are compared with “normal values” before the pandemic (Magnavita et al., 2020a), making their results debatable.

Protecting the mental health of acHCPs is critical in such pandemic times (National Center For PTSD, 2020), and one strategy might be promoting their psychological resilience (Fuchs et al., 2020). Resilience is described as a flexible adaptation to stressful events and improved recovery from negative emotional experiences. In the context of anaesthesia and intensive care medicine, resilience was defined as the ability to manage the breadth, depth, intensity and chronicity of the work demands (Fisher et al., 2018; Kelly et al., 2020; Bozdag and Ergun, 2021). Resilient persons have been shown to recover, maintain, and optimise their psychological health in adversity (Bonanno et al., 2011; Colombo et al., 2020). Resilience is associated with desirable health outcomes for HCPs (Bonanno et al., 2011; Fisher et al., 2018; Bozdag and Ergun, 2021; Riehm et al., 2021).

Research has shown that resilience can act as a “buffer” during high periods of stress and is a protective factor against burnout and post-traumatic stress disorder (Arrogante and Aparicio-Zaldivar, 2017; Kelly et al., 2021). During pandemics, there is an opportunity to improve HCWs’ ability to cope with and manage stress by building their resilience as a complementary approach to the necessary systemic efforts required. Recent studies also demonstrated that increased resilience has been linked to less persistent thinking about COVID-19 and increased wellbeing (Skalski et al., 2021).

Cultivating social support, actively constructing meaning, believing in own abilities, and having positive expectations increase resilience (Fisher et al., 2018; Colombo et al., 2020; Bozdag and Ergun, 2021). However, evidence is lacking regarding the influence of resilience on mental health throughout the COVID-19 pandemic and its subsequent waves in high-risk specialities, like anaesthesia, intensive care and emergency medicine. Attaining such information might help develop tailored evidence-based resilience-promoting interventions (Heath et al., 2020; Pollock et al., 2020; Santarone et al., 2020; Bozdag and Ergun, 2021).

We aimed to examine the variations in the psychological health of acHCPs during the first year of the COVID-19 pandemic (demonstrated by changes in COVID-19–related anxiety, perceived vulnerability, depressiveness, and symptoms of psychological trauma) and establish its association with resilience levels, and compare psychological health in time between front-line and second-line HCPs.

## Materials and Methods

According to the Swiss Act for Human Research, the Bern Cantonal Ethics Committee waived the need for ethical approval for this observational study on 1 April 2020 (BASEC Nr. 2020–00355). All procedures for this investigation followed the Helsinki Declaration. The study was registered at the United Kingdom-based International Standard Randomised Controlled Trial (ISRCTN 13694948). The authors sent a link to possible study participants, including a detailed cover letter explaining the research project (Fuchs et al., 2020). Electronic informed consent to participate was obtained from all participants at the beginning of the survey. No incentives were offered. All involved researchers complied with the Data Protection Act and the Swiss Law for Human Research. Data generated by the research project is stored on the password-protected server of the Institute of Psychology, Department of Health Psychology and Behavioural Medicine at the University of Bern.

### Study Design

The study was launched as an online survey with a 64-item questionnaire on April 2, 2020, hosted at Qualtrics (Provo, UT, United States), with access limited to one response per device, initially accessible for 2 weeks. Collected data included participant characteristics (i.e., age, sex, place of residence, relationship status, parental status, profession) and six validated self-reporting questionnaires to assess resilience, work-related sense of coherence, development of COVID-19–related anxiety, perceived vulnerability, depressiveness, and psychological trauma symptomatology. Additionally, we asked participants to report on exposure to COVID-19 patients and if they had been infected with COVID-19, belonged to a COVID-19 high-risk group, or had had close contact with a high-risk person (Fuchs et al., 2020). Patients considered “risk group” for severe COVID-19 included all those reporting at least one of the following characteristics: being over the age of 65 years, having high blood pressure, having Diabetes, having a Cardiovascular disease or Chronic respiratory diseases, or reporting conditions and therapies that weaken the immune system or cancer (Cdc, 2022).

The survey link was distributed through social media (i.e., LinkedIn, Facebook, Twitter), using the “snowballing” sampling technique, with personal invitations from all authors sent *via* email. Several international medical societies mailed the survey link to their members. The people contacted were asked to distribute the survey further. All of the participants who completed the baseline questionnaire for April 2020 (baseline - T0) were invited to continue their participation in July 2020 (T1), October 2020 (T2), January 2021 (T3), and April 2021 (T4), with the same 64-item questionnaire made available to them for 2 weeks for each measurement period. As per protocol, it was planned to run from April 2020 to October 2020 (Fuchs et al., 2020), but as the pandemic continued, it was prolonged until April 2021.

### Participants

We included all acHCPs, previously defined in the study protocol (Fuchs et al., 2020) aged over 18 years who worked full-time or part-time and agreed to participate. Study participants were asked if they worked as front-line medical HCPs (directly contacting COVID-19–infected patients i.e., if they diagnosed, treated, or provided care for COVID-19 patients) or second-line HCPs (i.e., with no direct contact with COVID-19 patients).

### Measurements

According to the previously published study protocol, the primary outcome of this study is the variation in COVID-19 anxiety in different regions, over three time periods, measured using a modified version of the Swine Influenza Anxiety Items, Wheaton et al. (2011), Fuchs et al. (2020) a 10-item survey developed to measure anxiety disorders and somatisation (Cronbach’s alpha = 0.85). The secondary outcomes included the measurement of *Perceived vulnerability* according to the Perceived Vulnerability to Disease questionnaire score (Duncan et al., 2009), a 15-item tool used to measure subjective vulnerability to disease (Cronbach’s alpha = 0.82); the Patient Health Questionnaire score, (Kroenke et al., 2001), a 9-item tool developed for depression evaluation (Cronbach’s alpha = 0.89); the Impact of Event Scale-6 score, (Thoresen et al., 2010), a 6-item tool for evaluation of symptoms of post-traumatic stress reactions (Cronbach’s alpha = 0.80); We used the Connor-Davidson Resilience Scale (CD-RISC-10) score (Campbell-Sills and Stein, 2007), a 10-item tool which is a short version of the CD-RISC-25 (Connor and Davidson, 2003) to evaluate individual resilience (Cronbach’s alpha = 0.85). All scales were validated for the English language, which was the language used in the questionnaire.

We assumed that the participants differed in their baseline level of resilience by measuring CD-RISC-10 (Campbell-Sills and Stein, 2007). To encounter this assumption, we calculated the baseline CD-RISC-10 for all participants and analysed it for normal distribution. We then grouped the participants into the three categories according to their CD-RISC-10 score within the normal distribution: “average level” = participants with baseline CD-RISC-10 in the range of mean value; “high level” = participants with baseline CD-RISC-10 in the range of mean value +1SD; “low level” = participants with baseline CD-RISC-10 in the range of mean value -1SD.

### Statistical Analysis

The required sample size was calculated using an *a priori* power analysis (G*Power V.3.1.42^1^). Assuming a small effect size (*f*^2^ = 0.15) for a repeated-measure analysis of variance with five-time points and within–between interactions (α = 0.05, 1-β = 0.80), the minimum required sample size was *n* = 69. According to the United Nations standard area codes for statistical use, participants were grouped in different world regions (United Nations [UN], 1999).

Statistical analysis was performed with R statistical language (R Core Team, 2021), and the packages *nlme, reghelper*, and *emmeans* to account for the hierarchical structure of the data (Snijders and Bosker, 2012). Normal distributions of the outcome variables were confirmed by inspection of the residual diagnostics of the fitted models using the R package *DHARMa.* Continuous predictors were mean-centred to reduce any multicollinearity (Snijders and Bosker, 2012). Restricted maximum likelihood (REML) was used for parameter estimation to reduce bias in estimates of variance and covariance parameters (Snijders and Bosker, 2012).

For each primary and secondary outcome variable, four different multilevel models were calculated. The initial model was a null (intercept-only) model for the inter-correlation coefficient (ICC), to determine whether a three-level model with participants grouped to different world regions (United Nations [UN], 1999) as the third level significantly improved the model fit. The three further models, in the final analysis, were: model 1, a non-linear unconditional growth model, to examine the within-participant trajectories of the cubic change across the time points; model 2, a conditional growth model with cross-level interaction, which included all of the predictor variables and a two-way cross-level interaction of resilience and time point, to examine the effects of resilience levels on the outcome over time; and model 3, a three-way cross-level interaction of resilience levels, time points and front-line and second-line H.C.P.s (i.e., a conditional growth model with three-way cross-level interactions), to examine differences in the slopes in terms of resilience levels between front-line and second-line HCPs. For the comparisons of the different models, the Akaike information criterion (AIC) and the Bayesian information criterion (BIC) were calculated (Snijders and Bosker, 2012). All of the models were significantly improved by including random intercepts and slopes.

## Results

### Demographics

**FIGURE 1:**
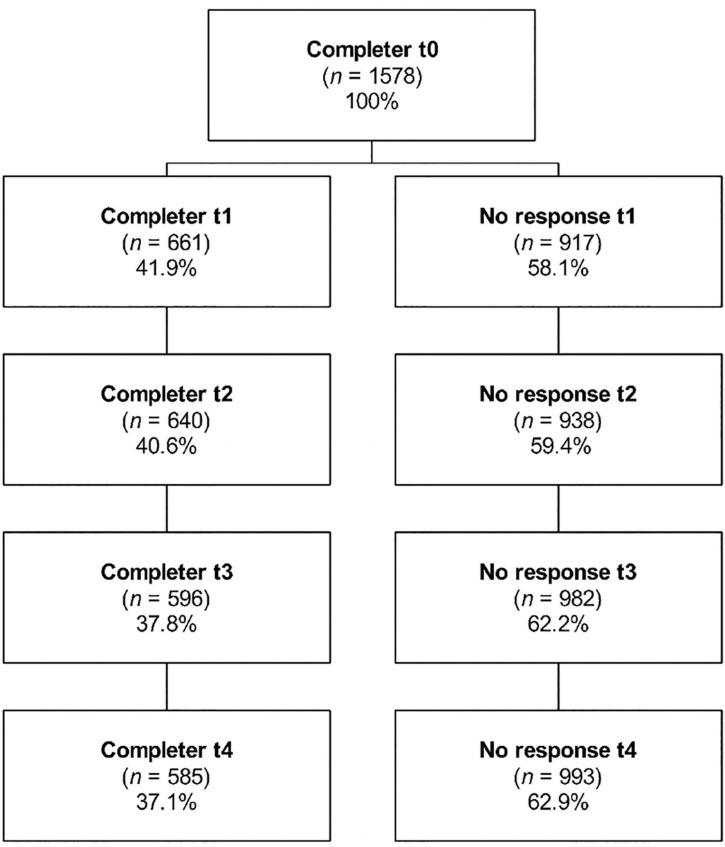
Study flowchart. April 2020 (baseline -T0), July 2020 (T1), October 2020 (T2), January 2021 (T3), and April 2021 (T4). All T0 participants were contacted in the remaining four rounds and we accepted for analysis participants who responded at least in four of the five time-points. We did not exclude participants that had not replied to the survey in previous rounds.

In April 2020, 1578 HCPs participated in the survey. Two-thirds of surveyed participants worked in anaesthesia, intensive care or in the emergency room. Of the initial cohort, 520 completed at least four out of the five surveys and were thus included in the analysis (response rate, 33.0%, Figure 1). HCPs across the globe participated in the survey; however, most of these HCPs were European (western, *n* = 258; 49.6%; southern, *n* = 112; 21.5%; northern, *n* = 80, 15.4%) (Table 1) – as per protocol, the demographic results of this cohort have been published previously (Berger-Estilita et al., 2022), as part of the data has been used for a different analysis. To address potential attrition bias, the HCPs included in the analysis were compared with those not further analysed due to less than four-time participation (Table 1, completers vs. non-completers). The study included significantly more HCPs infected with COVID-19 (more physicians and HCPs in western Europe, Table 1).

**TABLE 1:** Characteristics of the healthcare professionals included in this study.

Characteristic	Completers*	Non-completers	Statistics		
			*t (df)*	*(df)*	*p* ^†^
Total	520	1058			
**Age (years)**					
Mean (SD)	41.55 (10.69)	40.11 (10.38)	**2.53** *(1001)*	**0.21** *(2)*	**0.01**
**Gender [*n* (%)]**					
Female	321 (61.7)	661 (62.5)			**0.9**
Male	198 (38.1)	394 (37.2)			
**Risk group [*n* (%)]**					
No	454 (87.3)	897 (84.8)		**1.81** *(1)*	**0.2**
Yes	66 (12.7)	161 (15.2)			
**Occupation [*n* (%)]**					
Nurse	88 (16.9)	243 (23.0)		**8.87** *(2)*	**0.01**
Physician	364 (70.0)	667 (63.0)			
Other	68 (13.1)	148 (14.0)			
**Workplace [*n* (%)]**					
ICU	117 (22.5)	269 (25.4)		**4.11** *(4)*	**0.39**
Anaesthesia	184 (35.4)	396 (37.4)			
Emergency room	37 (7.1)	71 (6.7)			
Ward	38 (7.3)	63 (6.0)			
Other	144 (27.7)	259 (24.5)			
**Work status [*n* (%)]**					
Front-line	334 (64.2)	688 (65.0)		**0.75** *(1)*	**0.78**
Second-line	186 (35.8)	370 (35.0)			
**Contact with COVID-19 patients during the study [*n* (%)]**					
No	28 (5.4)	436 (41.2)		**215.56** *(1)*	**0**
Yes	492 (94.6)	622 (58.8)			
No	81 (15.6)	162 (15.3)		**0.02** *(1)*	**0.89**
Yes	439 (84.4)	896 (84.7)			
**Household [*n* (%)]**					
Live alone	78 (15.0)	185 (17.5)		**1.55** *(1)*	**0.21**
Live with someone	442 (85.0)	873 (82.5)			
**Children [*n* (%)]**					
No	240 (46.2)	511 (48.3)		**0.64** *(1)*	**0.42**
Yes	280 (53.8)	547 (51.7)			
**Infected with COVID-19 during study [*n* (%)]**					
No	232 (44.6)	599 (56.6)		**63.95** *(2)*	**0**
Yes	67 (12.9)	32 (3.00)			
Don’t know	221 (42.5)	427 (40.4)			
**World region [*n* (%)]**					
Western Europe	258 (49.6)	450 (42.5)		**12.27** *(4)*	**0.02**
Southern Europe	112 (21.5)	266 (25.1)			
Northern Europe	80 (15.4)	145 (13.7)			
North America	43 (8.3)	113 (10.7)			
Other regions	27 (5.2)	84 (7.9)			
**Resilience (CD-RISC-10) [*n* (%)]**					
Low (–1SD)	81 (15.6)	186 (17.6)		**1.51** *(2)*	**0.47**
Average	364 (70.0)	709 (67.0)			
High (+1SD)	75 (14.4)	163 (15.4)			

*Values are mean (SD) or number (proportion).*

*Participants with minimum of four out of five measurement points.

*^†^Comparisons between completers and non-completers (two-sided Welch’s t-tests for continuous data; Pearson’s Chi-squared tests for categorical variables).*

*CD-RISC-10, Connor-Davidson Resilience Scale; ICU, intensive care unit; SD standard deviation.*

### Primary Outcome: Predictors of COVID-19–Related Anxiety

**FIGURE 2:**
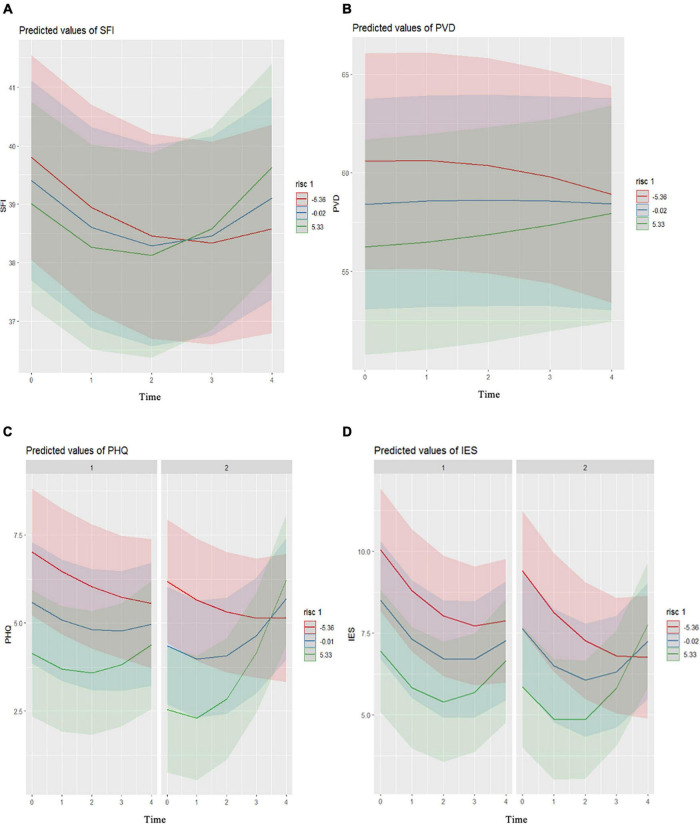
Trajectories of the mental health of the healthcare professionals across the five measurement points for their resilience levels (red, –1SD; blue, mean; green, + 1SD). Slopes of COVID-19–related anxiety **(A)**, perceived vulnerability **(B)**, depressiveness **(C)**, and psychological trauma symptomatology **(D)**, all by Resilience levels. According to front-line (c1,d1) and second-line (c2,d2) healthcare professionals. Time: 0, April 2020; 1, July 2020; 2, October 2020; 3, January 2021; 4, April 2021. S.F.I., Swine Influenza Anxiety Index; PVD, Perceived Vulnerability to Disease Scale; PHQ, Patient Health Questionnaire; IES, Impact of Event Scale; RISC, Connor-Davidson Resilience Scale.

Model 1 for COVID-19–related anxiety revealed a significantly improved fit for the inclusion of a “u-shaped” cubic trajectory (AIC = 13989.7; BIC = 14047.4, *p* = 0.001) with a significant relationship between time and COVID-19–related anxiety (*b* = 0.235, SE = 0.053, *p* < 0.001, Supplementary Table 1). Model 2 showed a significant negative relationship of interaction time and Resilience on COVID-19–related anxiety (*b* = 0.011, SE = 0.003, *p* < 0.001, slopes of HCP resilience, Figure 2A). *Post hoc* analysis revealed that all of these slopes differed significantly from each other (*p* = 0.002), and thus these trajectories of COVID-19–related anxiety differed between the different resilience groups. HCPs who reported close contact with people in the COVID-19 risk group showed considerably higher degrees of COVID-19–related anxiety than those who did not (*b* = 1.98, SE = 0.690, *p* = 0.004). Front-line and second-line HCP status was not a significant predictor of COVID-19–related anxiety (*p* = 0.150). The model explained 52.9% of the variance. In model 3, the added 3-way interaction was not significant (*p* = 0.194, Supplementary Table 1), which indicated no significant moderating effects of front-line and second-line HCPs on the Resilience–time interaction.

### Secondary Outcomes

#### Predictors of Perceived Vulnerability to Disease

Model 1 for Perceived vulnerability to disease (PVD) showed a significantly improved fit for the inclusion of fit for the inclusion of a “u-shaped” cubic trajectory (AIC = 18700.9; BIC = 18718.2, *p* < 0.001), with no significant relationship between time and perceived vulnerability to disease (*b* = –0.052, SE = 0.148, *p* = 0.726, Supplementary Table 2). The significant time–resilience interaction on PVD indicated that the “u-shaped” trajectory of PVD was influenced by resilience (Model 2, *b* = 0.020, SE = 0.0008, *p* = 0.016). The slopes by resilience are shown in Figure 2B. *Post hoc* analysis revealed that all of these slopes differed significantly from each other (*p* = 0.002). Increased age (*b* = 0.125, SE = 0.052, *p* = 0.016) and not belonging to the COVID-19 risk group (*b* = -2.92, SE = 1.40, *p* = 0.038) were associated with lower PVD. Front-line and second-line H.C.P. was not a significant predictor of PVD (*p* = 0.469). The model explained 72.0% of the variance. The added three-way interaction indicated no significant differences in the trajectories of perceived vulnerability to disease between the front-line and second-line HCPs (*p* = 0.378; model 3, Supplementary Table 1).

#### Predictors of Depressiveness

The total mean PHQ-9 score in April 2020 was 5.6 ± 5.1, in July 2020, 4.7 ± 4.6, in October 2020, 5.0 ± 4.9, in January 2021, 5.3 ± 5.0, and in April 2021, 5.4 ± 5.4. These data indicated mild depressiveness on average across the sample over time. Over the study period, 4.9% (*n* = 22) to 7.1% (*n* = 37) of HCPs reported severe depressiveness, 8.5% (*n* = 38) to 12.9% (*n* = 67) reported moderate depressiveness, and 26.5% (*n* = 124) to 30.4 (*n* = 144) reported mild depressiveness (Supplementary Table 3, proportion of participants with clinically relevant Major Depression across measurement points).

Model 1 for depressiveness revealed a significantly improved fit for the inclusion of a “u-shaped” trajectory (AIC = 13996.5; BIC = 14013.8, *p* = 0.003), with a significant relationship between time and depressiveness (*b* = 0.157 SE = 0.053, *p* = 0.003, Supplementary Table 4). Model 3 showed a significant relationship for the interaction of time with Figure 2B resilience and front-line and second-line HCP on depressiveness (*b* = 0.018, SE = 0.006, *p* = 0.006, Figure 2C). *Post hoc* analysis revealed significantly different trajectories of the high resilience front-line and second-line HCPs (*p* < 0.001) and significant differences between the average Resilience of the front-line and second-line HCPs (*p* = 0.004) (Supplementary Table 5).

Females (*b* = 1.09, SE = 0.308, *p* < 0.001) and HCPs who did not know their COVID-19 infection status (*b* = 0.187, SE = 0.077, *p* = 0.016) were more resilient. The HCPs who did not belong to the COVID-19 risk group showed lower degrees of depressiveness (*b* = –1.17, SE = 0.453, *p* = 0.010). Model 3 explained 59.0% of the variance.

#### Predictors of Psychological Trauma Symptomatology

The total mean IES-6 score in April 2020 was 9.2 ± 5.2, in July 2020, 6.8 ± 5.4, in October 2020, 7.2 ± 5.3, in January 2021, 8.0 ± 5.7, and in April 2021, 7.5 ± 5.3. These data indicate no clinically relevant trauma symptomatology on average across the whole sample during the study. Clinically relevant psychological trauma symptoms were reported by 25.7% (*n* = 114) to 46.4% (*n* = 241) of the HCPs during the study (Supplementary Table 3, proportion of participants with clinically relevant post-traumatic stress disorder across measurement points).

Model 1 for psychological trauma symptomatology revealed a significantly improved fit for the inclusion of a “u-shaped” trajectory (AIC = 14121.9; BIC = 14179.6, *p* < 0.001), with a significant relationship between time and psychological trauma symptomatology (*b* = 0.312, SE = 0.056, *p* < 0.001, Supplementary Table 6). Model 3 showed a significant relationship for the interaction of time with resilience and front-line and second-line HCP on psychological trauma symptomatology (*b* = 0.016, SE = 0.007, *p* = 0.018, Supplementary Table 6). The slopes of the psychological trauma symptomology by resilience for front-line and second-line HCPs are shown in Figure 2D. *Post hoc* analysis revealed that the slopes by degree of resilience did not differ significantly between front-line and second-line HCP (Supplementary Table 7). A higher degree of psychological trauma symptomatology was shown for females (*b* = 0.665, SE = 0.319, *p* = 0.037) compared to males, for young age (*b* = -0.036, *SE* = 0.017, *p* = 0.039), and for the HCPs who did not know their COVID-19 infection status (*b* = 0.171, SE = 0.080, *p* = 0.034). Model 3 explained 55.0% of the variance.

## Discussion

In this 1-year longitudinal observational study, individual resilience significantly influenced the trajectories of all outcome variables, similar to earlier reports (Bendau et al., 2021; Maekelae et al., 2021; Riehm et al., 2021). However, in contrast to studies with shorter follow-ups (Bendau et al., 2021; Maekelae et al., 2021), we found U-shaped trajectories for mental-health symptoms studied, which have not been described previously. HCPs with lower resilience reported worse mental health near the beginning of the pandemic in April 2020.

For HCPs with lower resilience (–1SD), COVID-19–related anxiety decreased from April 2020 to January 2021, and then steeply increased until April 2021, which might be explained by the rather small first COVID-19 wave (SARS-CoV-2 wildtype variant) with a lower death rate than expected, in contrast the huge second COVID-19 (SARS-CoV-2 delta variant) wave in fall/winter 2020 which led to many deaths all over the world. The HCPs with high degrees of resilience (+1SD) followed a similar pattern, although they showed their lowest anxiety in October 2020 and a steeper increase until April 2021 (Figure 1). This pattern was also seen for perceived vulnerability to disease. High resilience individuals are known to experience more significant symptom improvements or report more stable psychological health during stressful events (Bonanno et al., 2011; Riehm et al., 2021). In our sample, as low resilient individuals experienced more significant COVID-19-related threat during the early stages of the pandemic, improvements in their psychological symptoms might have been more notorious over time (Bendau et al., 2021). The unpredictable development of the COVID-19 pandemic might also come as a resilience-promoting factor for them. Individuals with higher degrees of resilience tend to attribute outcomes of events to being under their control (known as “internal locus of control”) (Thompson, 2021) and to positively predict their future emotional state (“biassed positive affective forecasting”) (Colombo et al., 2020). With time, the pandemic became more unpredictable in duration and progression, which might have had a more negative impact on HCPs showing greater resilience (Fisher et al., 2018; Colombo et al., 2020). In contrast, individuals with a lower degree of resilience, with a less biassed outlook and stronger beliefs in unpredictability, might react in the later stages with less frustration (Colombo et al., 2020).

In addition, people with high resilience might overestimate their coping abilities or underestimate the levels of distress they can experience in response to a potential psychological hazard (Bonanno et al., 2011; Thompson, 2021). This phenomenon has been described for HCPs (Backus et al., 2021). Such loss of resilience-promoting factors (i.e., social support, coping abilities, positive outlook) (Colombo et al., 2020; Bozdag and Ergun, 2021) can cause substantial depletion of coping resources in individuals with high degrees of resilience and with a strong internal locus of control (Thompson, 2021). These arguments align with our findings. The HCPs with high resilience lived more frequently alone and were not in a relationship, which might come as an extra burden in times of compulsory limited social contact, physical distancing, and curfews. We also hypothesise that the HCPs with high resilience might take on more shifts, take fewer breaks, and be given less team and organisational support, which might have been related to more significant strain on their mental health during the COVID-19 pandemic (Bonanno et al., 2011; Kisely et al., 2020; Bozdag and Ergun, 2021; Rieckert et al., 2021). Such hypotheses need to be confirmed in further studies.

Another novel result in the study is the different trajectories of depressiveness seen between the high resilience front-line and second-line HCPs. High and average resilience second-line HCPs showed steeper increases for their degree of depressiveness compared to high and average resilience front-line HCPs. A similar pattern emerged for psychological trauma symptomatology, although this did not reach significance in the *post hoc* analysis, probably due to a lack of power for such subgroup analyses. We hypothesise that such variations in time may be secondary to COVID-19-related changes at work, school, or in children’s education, COVID-19-related financial losses, fears about the future, added to social isolation and lockdowns, followed by some degree of adjustment and coping after the initial stress of the pandemic (Pieh et al., 2021; Van Zyl et al., 2021; Hampshire et al., 2022).

Front-line HCPs – mostly anaesthetists, intensivists, or emergency medicine providers – also have direct contact with infected patients, but to date, the effects of this contact on their mental health remained unclear. Some reports have described direct contact as a risk factor for worse mental health during novel viral and COVID-19 outbreaks (Kisely et al., 2020; Preti et al., 2020; Berger-Estilita et al., 2022), although some studies did not see such effects (Roberts et al., 2021). In line with our findings, one study reported trends toward lower burnout in Intensive Care Unit workers and less general psychological distress in front-line Emergency Department HCPs (Backus et al., 2021). Interestingly, the degree of resilience of the HCPs in our study was comparable to those of the general US population (Campbell-Sills and Stein, 2007), even though 5–7% of the HCPs reported clinically relevant worsening of symptoms of depressiveness, and 26–46% reported worsened psychological trauma symptomatology, with a peak in April 2020.

Our findings are relevant as resilience can be supported through tailored evidence-based interventions on personal and organisational levels (Heath et al., 2020; Pollock et al., 2020; Santarone et al., 2020; Bozdag and Ergun, 2021). The effectiveness of such interventions depends not only on the precise nature and goal of the intervention but also on the appropriate timing (Fisher et al., 2018). Additionally, decisions on the interventions for specific target groups should consider the risk factors. We found that younger age, belonging to the COVID-19 risk group or having close contact with a person belonging to a COVID-19 risk group, being a female, and showing insecurity about their current infection status are population subgroups that should be particularly targeted. While there some of the mentioned risk factors can’t be addressed by interventions – like being female or being young – our results indicate that specific types of individuals could be offered specific preventive strategies. A recent review summarises the types of interventions that could be implemented, which include fostering post-traumatic growth, resilience and adaptive coping (Finstad et al., 2021). On an organisational level, such strategies include counselling services, social connection strategies and targeted training. The latter may be complemented by peer-support, self-care, small-group discussions and mindfulness (Brooks et al., 2020). We, therefore, suggest an early screening of HCWs, with the aim of identifying individuals with low resilience and improving their mental health early.

The strengths of this study include its longitudinal design over 12 months, the use of validated questionnaires, and the advanced high-level statistical methods used to evaluate mental health trajectories and address the hierarchical structure and the non-linear nature of the longitudinal data. Our results bring new insights about significant psychological impacts on HCPs during the different waves of the pandemic. They identify specific resources that can be used to buffer the long-term effects of increased demands on HCPs.

Our study has limitations. We cannot exclude response bias (i.e., social desirability) and considerable regional variations of waves in the COVID-19 pandemic over the 12 months of the study. However, this did not interfere with the statistical models. We included only baseline resilience to keep the complexity low and avoid overloading the models. However, our first mental health assessment in April 2020 cannot be considered a proper baseline, as no information about the mental health of these HCPs before the first report of COVID-19 at the end of 2019 is available. We cannot exclude that preexisting diagnoses of the HCPs influenced our results (Bendau et al., 2021). We also do not have data on whether, after the pandemic, the HCPs with higher resilience will return to their previous functioning because resilience is defined as a return to baseline functioning (Bonanno et al., 2011; Fisher et al., 2018), because the pandemic is ongoing. The study sample also poses concerns: (1) it contains HCPs from all over the globe, (2) those completing all measurements were more likely to have been in contact with COVID-19 patients during study, (3) we used a convenience sample, (4) only a third of participants completed all surveys, and (5) about one third of participants were second-line HCP which might skew the comparison with the first-line HCPs. All these factors likely introduced more bias and hamper generalizability of our conclusions. The COVID-19 evolution at the measured time points may have been distinct in different regions, underestimating our results. Finally, we have to acknowledge the limitations of the snowball sampling technique, particularly the unintentional overvalue of some groups in comparison to others, so representativeness of the sample is not guaranteed.

In conclusion, the trajectories for HCPs with high and low resilience differed significantly. Second-line H.C.P.s with high and average degrees of resilience showed steeper worsening of their depressiveness than front-line HCPs (like anaesthesia, intensive care, and emergency medicine providers) with high and average degrees of resilience. Our study indicates that resilience-enhancing interventions should be focused on HCPs with low resilience at the beginning of a pandemic. In contrast, HCPs with high resilience might benefit from resilience-enhancing interventions at the later phases. This would help buffer the adverse effects of the specific high demands on HCPs during a pandemic, indicating the need to incorporate temporal aspects.

## Data Availability Statement

The original contributions presented in this study are included in the article/Supplementary Material, further inquiries can be directed to the corresponding author.

## Ethics Statement

This investigation followed the Helsinki Declaration; all researchers complied with the Swiss Human Research Act. The Bern Cantonal Ethics Committee waived the need for ethical approval (Req-2020-00355, April 1, 2020). The patients/participants provided their written informed consent to participate in this study.

## Author Contributions

AF and RG had the idea of the study. AF, JB-E, and RG recruited the participants. SA collected and analysed the data. SA and RG interpreted the analysis and drafted the manuscript. All authors designed the study, critically revised the manuscript, and agreed to the final version publication.

## Conflict of Interest

The authors declare that the research was conducted in the absence of any commercial or financial relationships that could be construed as a potential conflict of interest.

## Publisher’s Note

All claims expressed in this article are solely those of the authors and do not necessarily represent those of their affiliated organizations, or those of the publisher, the editors and the reviewers. Any product that may be evaluated in this article, or claim that may be made by its manufacturer, is not guaranteed or endorsed by the publisher.

## Footnotes

1
gpower.hhu.de/
