# Erratum: COVID-19–Related trajectories of psychological health of acute care healthcare professionals: A 12-month longitudinal observational study

**DOI:** 10.3389/fpsyg.2023.1180634

**Published:** 2023-03-17

**Authors:** Frontiers Production Office

**Affiliations:** Frontiers Media SA, Lausanne, Switzerland

**Keywords:** COVID-19, acute care, psychological resilience, healthcare workers, mental health

An omission to the funding section of the original article was made in error. The following sentence has been added: “Open access funding was provided by the University of Bern.”

The original article has been updated.

