# Supplementary material for: COVID-19–Related Trajectories of Psychological Health of Acute Care Healthcare Professionals: A 12-Month Longitudinal Observational Study
Source: Front Psychol. 2022 Jun 30;13:900303. doi: 10.3389/fpsyg.2022.900303 (PMC9280365; doi:10.3389/fpsyg.2022.900303)
Supplement: Supplementary file 1 [file Table_1.docx]

**Table S1.** Results of the multilevel analysis for COVID-19–related anxiety of the healthcare professionals (n = 520; observations = 2372).

| **Analysis** | **Variables** | **Model** | | | | | | |
| --- | --- | --- | --- | --- | --- | --- | --- | --- |
|  |  | **1** | | **2** | | **3** | | |
|  |  | Unconditional cubic growth | | Conditional cubic growth, 2-way cross-level interaction | | Conditional cubic growth, 3-way cross-level interaction | | |
|  |  | ***b*** | **SE** | ***b*** | **SE** | ***b*** | **SE** |  |
| Fixed effects | Intercept | 40.756*** | 0.221 | 39.351*** | 0.924 | 39.320*** | 0.930 |  |
| Level I | Time | -1.019*** | 0.216 | -1.037*** | 0.211 | -1.037*** | 0.212 |  |
|  | (Time)^2^ | 0.235*** | 0.053 | 0.241*** | 0.058 | 0.244*** | 0.060 |  |
| Level II | Female |  |  | 0.293 | 0.305 | 0.293 | 0.304 |  |
|  | Age |  |  | -0.001 | 0.016 | -0.001 | 0.016 |  |
|  | No-risk population |  |  | -0.320 | 0.447 | 0.053 | 0.446 |  |
|  | No children |  |  | -0.424 | 0.356 | -0.422 | 0.335 |  |
|  | Live alone |  |  | -0.279 | 0.477 | -0.282 | 0.476 |  |
|  | Contact with risk population |  |  | 1.982** | 0.690 | 1.985** | 0.690 |  |
|  | No relationship |  |  | -0.045 | 0.453 | 0.053 | 0.453 |  |
|  | Infected during study |  |  | 0.028 | 0.076 | 0.027 | 0.076 |  |
|  | Second-line HCP |  |  | -0.456 | 0.316 | -0.421 | 0.416 |  |
|  | Workplace |  |  | -0.001 | 0.944 | 0.000 | 0.094 |  |
|  | Resilience |  |  | -0.074* | 0.036 | -0.042 | 0.047 |  |
| Cross-level | (Time)^2^*Resilience |  |  | 0.011*** | 0.003 | 0.007 | 0.004 |  |
|  | (Time)^2^*Second-line HCP |  |  |  |  | -0.005 | 0.037 |  |
|  | Front-line HCP*Resilience |  |  |  |  | -0.077 | 0.073 |  |
|  | (Time)^2^*Resilience*Second-line HCP |  |  |  |  | 0.009 | 0.007 |  |
| Variance components | | **Estimate** | | **Estimate** | | **Estimate** | | |
| Within participants (Level 1)  Between participants (Level 2)  Slope variance (Time)  Slope variance (Time)^2^ | | 12.79 | | 12.80 | | 12.81 | | |
|  |  | 8.56 | | 8.01 | | 7.92 | | |
|  |  | 6.02  0.74 | | 6.03  0.72 | | 6.19  0.74 | | |

* *p* <0.05; ** *p* <0.01; *** *p* <0.001

HCP, healthcare professional; SE, standard error
