# Supplementary material for: COVID-19–Related Trajectories of Psychological Health of Acute Care Healthcare Professionals: A 12-Month Longitudinal Observational Study
Source: Front Psychol. 2022 Jun 30;13:900303. doi: 10.3389/fpsyg.2022.900303 (PMC9280365; doi:10.3389/fpsyg.2022.900303)
Supplement: Supplementary file 2 [file Table_2.docx]

**Table S2.** Results of the multilevel analysis for perceived vulnerability to COVID-19 of the healthcare professionals (n = 520; observations = 2368).

| **Analysis** | **Variables** | **Model** | | | | | | |
| --- | --- | --- | --- | --- | --- | --- | --- | --- |
|  |  | **1** | | **2** | | **3** | | |
|  |  | Unconditional cubic growth | | Conditional cubic growth, 2-way cross-level interaction | | Conditional cubic growth, 3-way cross-level interaction | | |
|  |  | ***b*** | **SE** | ***b*** | **SE** | ***b*** | **SE** |  |
| Fixed effects | Intercept | 54.62*** | 0.592 | 58.09*** | 2.89 | 58.28*** | 2.90 |  |
| Level I | Time | 0.210 | 0.507 | 0.202 | 0.508 | 0.201 | 0.509 |  |
|  | (Time)^2^ | -0.052 | 0.149 | -0.050 | 0.148 | -0.619 | 0.151 |  |
| Level II | Female |  |  | 1.651 | 0.954 | 1.642* | 0.953 |  |
|  | Age |  |  | -0.125* | 0.052 | -0.125* | 0.052 |  |
|  | No-risk population |  |  | -2.923* | 1.401 | -2.947* | 1.420 |  |
|  | No children |  |  | -1.850 | 1.114 | -1.786 | 1.114 |  |
|  | Live alone |  |  | 0.079 | 1.417 | 1.241 | 1.493 |  |
|  | Contact with risk population |  |  | -1.873 | 2.157 | -1.975 | 2.158 |  |
|  | Relationship |  |  | 0.079 | 1.417 | 0.207 | 1.420 |  |
|  | Infected during study |  |  | 0.280 | 0.239 | 0.248 | 0.240 |  |
|  | Second-line HCP |  |  | 0.718 | 0.991 | 0.400 | 1.267 |  |
|  | Workplace |  |  | -0.108 | 0.296 | -0.088 | 0.296 |  |
|  | Resilience |  |  | -0.406*** | 0109 | -0.368** | 0.143 |  |
| Cross-level | (Time)^2^*Resilience |  |  | 0.020* | 0.008 | 0.026*** | 0.011 |  |
|  | (Time)^2^*Second-line HCP |  |  |  |  | 0.281 | 0.093 |  |
|  | Front-line HCP*Resilience |  |  |  |  | -0.099 | 0.222 |  |
|  | (Time)^2^*Resilience*Second-line HCP |  |  |  |  | -0.015 | 0.018 |  |
| Variance components | | **Estimate** | | **Estimate** | | **Estimate** | |  |
| Within participants (Level 1)  Between participants (Level 2)  Slope variance (Time)  Slope variance (Time)^2^ | | 58.85 | | 58.68 | | 58.66 | |  |
|  |  | 128.83 | | 121.71 | | 122.22 | |  |
|  |  | 53.55 | | 54.14 | | 54.11 | |  |
|  |  | 6.58 | | 6.49 | | 6.49 | |  |

* *p* <0.05; ** *p* <0.01; *** *p* <0.001

HCP, healthcare professional; SE, standard error
