# Supplementary material for: COVID-19–Related Trajectories of Psychological Health of Acute Care Healthcare Professionals: A 12-Month Longitudinal Observational Study
Source: Front Psychol. 2022 Jun 30;13:900303. doi: 10.3389/fpsyg.2022.900303 (PMC9280365; doi:10.3389/fpsyg.2022.900303)
Supplement: Supplementary file 3 [file Table_3.docx]

**Table S3.** Frequencies of clinical relevant Major Depression and Posttraumatic Stress Disorder (PTSD) across measurement points

|  |  | **T1**  *April 2020* *n* = 520 | |  | | | **T2**  *July 2020 n* = 445 | | **T3**  *October 2020* *n* = 468 | | | | | **T4**  *January 2021*  *n* = 473 | | | **T5**  *April 2021*  *n* = 466 | | |
| --- | --- | --- | --- | --- | --- | --- | --- | --- | --- | --- | --- | --- | --- | --- | --- | --- | --- | --- | --- |
|  |  | ***N*** | ***%*** | |  | ***N*** | | ***%*** |  | ***N*** | ***%*** |  | ***N*** | | ***%*** |  | | ***N*** | ***%*** |
| Major Depression (PHQ-9) | |  |  | |  |  | |  |  |  |  |  |  | |  |  | |  |  |
|  | Severe | 37 | 7.1 | |  | 22 | | 4.9 |  | 25 | 5.3 |  | 29 | | 6.2 |  | | 32 | 6.9 |
|  | Moderate | 67 | 12.9 | |  | 38 | | 8.6 |  | 48 | 10.3 |  | 45 | | 9.5 |  | | 48 | 10.3 |
|  | Mild | 144 | 27.7 | |  | 130 | | 29.2 |  | 124 | 26.5 |  | 144 | | 30.4 |  | | 132 | 28.3 |
|  | No | 272 | 52.3 | |  | 255 | | 57.3 |  | 271 | 57.9 |  | 255 | | 53.9 |  | | 254 | 54.5 |
|  |  |  |  | |  |  | |  |  |  |  |  |  | |  |  | |  |  |
| Posttraumatic Stress Disorder (IES-6) | |  |  | |  |  | |  |  |  |  |  |  | |  |  | |  |  |
|  | PTSD-Symptoms | 241 | 46.4 | |  | 114 | | 25.7 |  | 142 | 30.3 |  | 179 | | 37.8 |  | | 147 | 31.6 |
|  | Not relevant PTSD-Symptoms | 279 | 53.6 | |  | 330 | | 74.3 |  | 326 | 69.7 |  | 294 | | 62.2 |  | | 319 | 68.4 |
