# Supplementary material for: COVID-19–Related Trajectories of Psychological Health of Acute Care Healthcare Professionals: A 12-Month Longitudinal Observational Study
Source: Front Psychol. 2022 Jun 30;13:900303. doi: 10.3389/fpsyg.2022.900303 (PMC9280365; doi:10.3389/fpsyg.2022.900303)
Supplement: Supplementary file 4 [file Table_4.docx]

**Table S4.** Results of the multilevel analysis for COVID-19–related depressiveness of the healthcare professionals (n = 520; observations = 2372).

| **Analysis** | **Variables** | **Model** | | | | | | |
| --- | --- | --- | --- | --- | --- | --- | --- | --- |
|  |  | **1** | | **2** | | **3** | | |
|  |  | Unconditional cubic growth | | Conditional cubic growth, 2-way cross-level interaction | | Conditional cubic growth, 3-way cross-level interaction | | |
|  |  | ***b*** | **SE** | ***b*** | **SE** | ***b*** | **SE** |  |
| Fixed effects | Intercept | 5.50*** | 0.211 | 4.94*** | 0.927 | 5.25*** | 0.934 |  |
| Level I | Time | -0.620*** | 0.186 | -0.616*** | 0.186 | -0.616*** | 0.186 |  |
|  | (Time)^2^ | 0.157** | 0.053 | 0.156** | 0.052 | 0.116* | 0.053 |  |
| Level II | Female |  |  | 1.090*** | 0.307 | 1.089*** | 0.308 |  |
|  | Age |  |  | -0.020 | 0.017 | -0.020 | 0.017 |  |
|  | No-risk population |  |  | -1.165** | 0.453 | -1.171** | 0.453 |  |
|  | No children |  |  | 0.161 | 0.359 | 0.150 | 0.360 |  |
|  | Live alone |  |  | -0.189 | 0.481 | -0.164 | 0.481 |  |
|  | Contact with risk population |  |  | 0.756 | 0.686 | 0.748 | 0.687 |  |
|  | Relationship |  |  | -0.313 | 0.456 | -0.349 | 0.186 |  |
|  | Infected during study |  |  | 0.180* | 0.077 | 0.186* | 0.077 |  |
|  | Second-line HCP |  |  | -0.373 | 0.320 | -1.219** | 0.408 |  |
|  | Workplace |  |  | -0.004 | 0.095 | -0.009 | 0.096 |  |
|  | Resilience |  |  | -0.292*** | 0.035 | -0.269 | 0.046 |  |
| Cross-level | (Time)^2^*Resilience |  |  | 0.016*** | 0.003 | 0.010* | 0.004 |  |
|  | (Time)^2^*Second-line HCP |  |  |  |  | 0.126*** | 0.035 |  |
|  | Front-line HCP*Resilience |  |  |  |  | -0.072 | 0.072 |  |
|  | (Time)^2^*Resilience*Second-line HCP |  |  |  |  | 0.018** | 0.006 |  |
| **Variance components** | | **Estimate** | | **Estimate** | | **Estimate** | |  |
| Within participants (Level 1)  Between participants (Level 2)  Slope variance  Slope variance (Time)^2^ | | 10.56 | | 10.50 | | 10.50 | |  |
|  |  | 13.56 | | 10.24 | | 10.16 | |  |
|  |  | 3.65 | | 3.78 | | 3.74 | |  |
|  |  | 0.57 | | 0.49 | | 0.55 | |  |

*, *p* <0.05; **, *p* <0.01; ***, *p* <0.001

HCP, healthcare professional; SE, standard error
