# Supplementary material for: COVID-19–Related Trajectories of Psychological Health of Acute Care Healthcare Professionals: A 12-Month Longitudinal Observational Study
Source: Front Psychol. 2022 Jun 30;13:900303. doi: 10.3389/fpsyg.2022.900303 (PMC9280365; doi:10.3389/fpsyg.2022.900303)
Supplement: Supplementary file 5 [file Table_5.docx]

**Table S5.** *Post-hoc-Contrasts of Resilience Levels between Front- and Secondliners in trajectories of depressiveness (PHQ-9)*

| **Comparisons** | **Estimate Difference** | ***SE*** | ***df Bound*** | ***t*** | ***p*** |
| --- | --- | --- | --- | --- | --- |
| Frontliner RISC (+1SD) vs Secondliner RISC (+1SD) | -0.885 | 0.199 | 513 | -4.45 | 0.001 |
| Frontliner RISC (0) vs Secondliner RISC (0) | -0.506 | 0.140 | 508 | -3.63 | 0.004 |
| Frontliner RISC (-1SD) vs. Secondliner RISC (-1SD) | -0.127 | 0.191 | 504 | -0.66 | 0.986 |

Note: n = 520; observations = 2372, comparisons adjusted by tukey for multiple testing
