# Supplementary material for: COVID-19–Related Trajectories of Psychological Health of Acute Care Healthcare Professionals: A 12-Month Longitudinal Observational Study
Source: Front Psychol. 2022 Jun 30;13:900303. doi: 10.3389/fpsyg.2022.900303 (PMC9280365; doi:10.3389/fpsyg.2022.900303)
Supplement: Supplementary file 6 [file Table_6.docx]

**Table S6.** Results of the multilevel analysis for COVID-19–related psychological trauma symptomatology of the healthcare professionals (n = 520; observations = 2371).

| **Analysis** | **Variables** | **Model** | | | | | |
| --- | --- | --- | --- | --- | --- | --- | --- |
|  |  | **1** | | **2** | | **3** | |
|  |  | Unconditional cubic growth | | Conditional cubic growth, 2-way cross-level interaction | | Conditional cubic growth, 3-way cross-level interaction | |
|  |  | ***b*** | **SE** | ***b*** | **SE** | ***b*** | **SE** |
| Fixed effects | Intercept | 8.84*** | 0.218 | 8.04*** | 0.966 | 8.16*** | 0.974 |
| Level I | Time | -1.489*** | 0.213 | -1.483*** | 0.213 | -1.483*** | 0.213 |
|  | (Time)^2^ | 0.312*** | 0.056 | 0.310*** | 0.058 | 0.294*** | 0.060 |
| Level II | Female |  |  | 0.665* | 0.319 | 0. 665* | 0.319 |
|  | Age |  |  | -0.036* | 0.017 | -0.036* | 0.017 |
|  | No-risk population |  |  | -0.676 | 0.469 | -0.674 | 0.469 |
|  | No children |  |  | -0.384 | 0.373 | -0.405 | 0.373 |
|  | Live alone |  |  | 0.243 | 0.499 | 0.272 | 0.500 |
|  | Contact with risk population |  |  | 1.094 | 0.719 | 1.115 | 0.720 |
|  | Relationship |  |  | -0.539 | 0.474 | -0.586 | 0.475 |
|  | Infected during study |  |  | 0.161* | 0.080 | 0.171* | 0.080 |
|  | Second-line HCP |  |  | -0.487 | 0.331 | -0.867* | 0.433 |
|  | Workplace |  |  | 0.099 | 0.015 | 0.008 | 0.099 |
|  | Resilience |  |  | -0.304*** | 0.037 | -0.289*** | 0.049 |
| Cross-level | (Time)^2^*Resilience |  |  | 0.017*** | 0.003 | 0.011** | 0.004 |
|  | (Time)^2^*Second-line HCP |  |  |  |  | 0.054 | 0.036 |
|  | Front-line HCP*Resilience |  |  |  |  | -0.043 | 0.076 |
|  | (Time)^2^*Resilience*Second-line HCP |  |  |  |  | 0.016* | 0.007 |
| **Variance components** | | **Estimate** | | **Estimate** | | **Estimate** | |
| Within participants (Level 1)  Between participants (Level 2)  Slope variance (Time)  Slope Variance (Time)^2^ | | 13.47 | | 13.45 | | 13.44 | |
|  |  | 12.61 | | 9.66 | | 9.70 | |
|  |  | 5.45 | | 5.50 | | 5.54 | |
|  |  | 0.70 | | 0.68 | | 0.68 | |

*, p <0.05; **, p <0.01; ***, p <0.001

HCP, healthcare professional; SE, standard error
